# The diagnosis of lipid transfer protein allergy—Discriminating between sensitisation and allergy

**DOI:** 10.1002/clt2.70065

**Published:** 2025-05-21

**Authors:** B. Olivieri, G. Scadding, I. J. Skypala

**Affiliations:** ^1^ Allergy Unit University Hospital of Verona Verona Italy; ^2^ Royal Brompton & Harefield Hospitals Part of Guys & St Thomas NHS Foundation Trust London UK; ^3^ Department of Allergy and Clinical Immunology National Heart and Lung Institute Imperial College London London UK

**Keywords:** diagnosis, food allergy, lipid transfer protein, Pru p 3, total IgE

## Abstract

**Background:**

Sensitisation to Lipid Transfer Proteins (LTP), usually ascertained by undertaking a test to the peach LTP allergen Pru p 3, is common but does not always indicate LTP allergy. Improving the diagnostic process would ensure the correct diagnosis and management of this complex condition.

**Objectives:**

To determine the diagnostic value of Pru p 3 and other LTP component allergens in UK adults.

**Methods:**

A retrospective review was undertaken of adults referred to the Allergy Unit at the Royal Brompton & Harefield Hospitals (RBHT) London (UK), between 2012 and 2022 who were sensitised to Pru p 3. Those with a final diagnosis of LTP allergy were compared to those sensitized to Pru p 3 but not diagnosed with LTP allergy.

**Results:**

Of 285 patients with a positive Pru p 3, 157 (55%) were diagnosed with LTP allergy. LTP allergic patients were more likely to have a higher level of Pru p 3, and a lower level of total IgE. The ratio of Pru p 3:total IgE was the most accurate diagnostic marker of LTP allergy, with a receiver operating characteristics AUC of 0.880. A diagnosis of LTP allergy was also significantly associated with sensitisation to the LTP in peanut (Ara h 9, *p* < 0.001), and hazelnut (Cor a 8, *p* < 0.001).

**Conclusion:**

Sensitisation to Pru p 3 may not always indicate an LTP allergy. Our data suggests that the Pru p 3:total IgE ratio, and sensitisation to Ara h 9 and Cor a 8 can support the diagnosis of LTP allergy in individuals sensitised to Pru p 3.

## 1 INTRODUCTION

Allergic reactions to plant foods are probably the most common manifestation of IgE‐mediated food allergy in adults. In the United Kingdom (UK), mild new‐onset allergic reactions to fruits, vegetables, nuts and legumes usually are a manifestation of Pollen Food Syndrome (PFS).^1^, ^2^ However, in recent years, another plant food allergy, lipid transfer protein (LTP) allergy, which is widespread in Mediterranean areas, has become an emerging problem in Northern European regions.^3^, ^4^, ^5^, ^6^, ^7^ In the UK, diagnosis can be complicated due to co‐sensitisation to pollens, and also because the common LTP food triggers could indicate a differential diagnosis of PFS or a primary allergy to tree nuts or peanuts. In polysensitised patients, it may be difficult to discriminate which are clinically relevant, leading to an incorrect diagnosis.^1^ Although Pru p 3, the peach LTP, is considered a reliable marker of sensitisation to LTP, it cannot discriminate between sensitisation and true allergy.^8^ Sensitisation to Pru p 3 is a common phenomenon in Spain and Italy, and individuals who are sensitised to LTP can be asymptomatic to LTP food triggers. Thus, a positive test may not always indicate that LTP allergy is the cause of one or all of the patient's reactions to plant foods.^9^, ^10^, ^11^

The concentration of Pru p 3 may be a good marker of diagnostic certainty, but studies have shown conflicting results with levels below the traditional cut‐off point of 0.35 kUA/L possibly still being significant.^12^, ^13^ Sensitisation to LTP other than Pru p 3 might also support a diagnosis of LTP allergy, such as the LTP in walnuts, peanuts, hazelnuts, and apple.^14^, ^15^ In addition to testing, it may be necessary to clarify test results with a food challenge (FC) as individuals with LTP allergy may only react to the food trigger in the presence of co‐factors such as exercise, non‐steroidal anti‐inflammatory drugs (NSAID) or alcohol.^16^ Although the FC is the gold standard for diagnosing food allergy,^17^ it has several limitations when applied to LTP allergy. It cannot confirm whether symptoms are specifically due to LTP sensitization, and it is difficult to standardize due to undefined threshold doses for plant foods and the risk of false negatives from low LTP content.^9^ Thus, LTP allergy represents a diagnostic challenge for the clinician. Therefore, the aim of this retrospective study was to identify the predictive value of skin prick tests (SPT), and component LTP allergens including Pru p 3, in the diagnostic workup of LTP allergy.

## 2 METHODS

Clinical data from adult patients referred for a suspected food allergy between 2012 and 2022 to the Allergy Unit at the Royal Brompton & Harefield NHS Foundation Trust (RBHT) in London (UK) were retrospectively analysed. All Adult subjects who had been tested to Pru p 3 were selected and those with a result ≥0.35 KUA/L were included in the analysis. The clinical records of all selected participants were then reviewed to ascertain the final diagnosis. The diagnosis, made by individual clinicians, included a clinical history containing details of the suspected trigger foods, foods avoided and consumed, reported symptoms, presence or absence of co‐factors, and co‐morbidities. They also undertook tests to the suspected trigger foods and aeroallergens, including Pru p 3, and where available/relevant, other LTP components, most usually to peanuts, walnuts and hazelnuts. However, the diagnosis did not follow a standardised protocol or involve a FC as it was considered that the history and sensitisation to LTP allergens was sufficient on which to make a diagnosis. The final diagnosis from the original patient record was documented as either having a diagnosis of LTP allergy (LTP allergic) or having an alternate diagnosis (LTP sensitised). In some instances, no diagnosis was made due to loss to follow‐up and these individuals were not included in the final analysis.

The data collected for all participants included allergic co‐morbidities, symptoms, foods involved, and results of allergy tests including skin prick (SPT) and/or specific IgE (sIgE) tests. SPTs were performed using standardized techniques according to international guidelines. The extracts included aeroallergens and foods (either from ALK Abelló, Horsholm, Denmark or from Stallergenes, Antony, France), and positive and negative control solutions (histamine hydrochloride 10 mg/mL and diluent). In some instances, testing with fresh foods was undertaken using the prick‐to‐prick test method; the intact food was pierced through the peel or skin with a sterile lancet (ALK Abelló) and then used to prick the skin of the subject. All SPTs were recorded as positive if the size of any resulting wheal was ≥3 mm greater than the negative control.^17^ Specific IgE was measured by ImmunoCAP (Thermo Fisher Scientific). Serum sIgE values were quantified in kilounits of allergen (kUA) per litre, with values ≥0.35 kUA/L considered positive.

Due to the retrospective nature of the review, other tests performed varied depending on the history. Therefore, in addition to results for Pru p 3, the rest of the data collected was limited to only those foods or aeroallergens considered relevant to the diagnosis of LTP allergy or an alternative diagnosis. These included total IgE, whole SPT/IgE extracts (almond, hazelnut, Macadamia nut, peanut, pistachio nut, cashew nut, Brazil nut, pecan, walnut, apple, tomato, silver birch pollen, grass pollen, mixed tree pollen, mugwort pollen and plane tree pollen) and component allergens (peach ‐ Pru p 3; hazelnut ‐ Cor a 14, Cor a 9, Cor a 8, Cor a 1; peanut ‐ Ara h 2, Ara h 9, Ara h 8; cashew nut ‐ Ana o 3; Brazil nut ‐ Ber e 1; walnut ‐ Jug r 1, Jug r 3; apple ‐ Mal d 1, Mal d 3). The retrospective nature of the study, and the fact that no patient‐identifiable data was seen by non‐clinical staff meant that ethical approval was not required, but the study was approved as an audit by the Royal Brompton & Harefield Hospital.

### 2.1 Statistical analysis

All computations were performed using the SPSS 29.0 statistical package (SPSS Inc., Chicago, IL). Distributions of continuous variables in groups were expressed as means ± standard deviations. Differences in quantitative variables were assessed for statistical significance by Student's *t*‐test. Qualitative data were analysed by the chi‐square test. Pearson or Spearman correlations were used to assess the association between two linear or non‐parametric variables, respectively. To assess diagnostic accuracy and establish the sensitivity and specificity of variables, receiver operating characteristics (ROC) curves were determined using as ‘state variable’ the category to which a subject belonged according to the clinical diagnosis. For any value of the considered measurement the corresponding values of sensitivity and specificity (plotted as 1‐specificity) could be immediately defined from the ROC curve. Area under the ROC curve with 95% confidence interval was also calculated. *p* values lower than 0.05 were considered statistically significant.

## 3 RESULTS

### 3.1 Demographic data

Of the 298 subjects with a positive Pru p 3, 157 (55%) received a diagnosis of LTP allergy (LTP allergic), whilst 128 (45%) received an alternative diagnosis (LTP sensitised) and 13 had an unknown/inconclusive diagnosis and were therefore not included in the analysis. Of the LTP‐allergic group, 64% were female compared to 56% of the LTP sensitised group (*p* = 0.165). The mean age of the LTP‐allergic group was 33.2 years at diagnosis compared to 33.6 years for the LTP sensitised group (*p* = 0.792).

### 3.2 LTP allergy diagnostic markers

The LTP‐allergic group had a greater mean level of Pru p 3 compared to the LTP sensitised group (*p* < 0.001) (Table 1). Although all patients were tested to Pru p 3, only 48% had a SPT with peach extract. Of these, a significantly greater number of LTP allergic patients had a positive SPT to peach (78/87% ‐ 84%) compared to the LTP sensitised group (18/57% ‐ 34%) (*p* < 0.001) (Table 2) The wheal diameter in those with a positive SPT (>3 mm) was 7.15 mm (3–16 mm) in the LTP allergic group compared to 5.88 mm (3–10 mm) in the LTP sensitised group (*p* = 0.047). There was also a significant difference in mean total IgE levels between the groups, being 1187 kUA/L (15‐43,017) in LTP allergic individuals compared to 2724 kUA/L (56‐47,893) in LTP sensitised patients (*p* = 0.0161) (Table 1). In order to confirm this significant difference, the values were re‐assessed to exclude individuals with a total IgE ≥10,000 and the difference between the two groups increased in significance (mean total IgE 624.38 LTP allergic v 1832.66 LTP sensitised, median 253 LTP allergic v 862 LTP sensitised) (*p* = <0.00001).

**TABLE 1:** Comparison of levels of total and specific IgE and components.

Allergen	Diagnosis	Mean	Median	Sig
Pru p 3	LTP allergic	12.5	6.24	*p* < 0.001
	LTP sensitised	4.54	1.19	
Total IgE	LTP allergic	1187.14	256.00	*p* = 0.007
	LTP sensitised	2724.66	983.00	
Ara h 2	LTP allergic	1.94	0.01	*p* = 0.001
	LTP sensitised	7.87	0.57	
Ara h 8	LTP allergic	5.77	0.15	*p* = 0.002
	LTP sensitised	19.08	6.92	
Ara h 9	LTP allergic	9.51	3.13	*p* < 0.001
	LTP sensitised	1.82	0.41	
Cor a 1	LTP allergic	18	0.92	*p* = 0.002
	LTP sensitised	42.08	20.10	
Cor a 8	LTP allergic	5.5	1.93	*p* = 0.003
	LTP sensitised	1.44	0.35	
Cor a 9	LTP allergic	0.47	0.04	*p* = 0.033
	LTP sensitised	1.04	0.23	
Cor a 14	LTP allergic	0.29	0.01	*p* = 0.057
	LTP sensitised	1.44	0.13	
Jug r 1	LTP allergic	0.49	0.01	*p* = 0.025
	LTP sensitised	4.45	0.22	
Jug r 3	LTP allergic	6.19	3.31	*p* = 0.069
	LTP sensitised	2.33	0.64	
Mugwort	LTP allergic	7.77	2.1	*p* = 0.311
	LTP sensitised	5.79	0.79	
Birch	LTP allergic	14.18	1.47	*p* = 0.005
	LTP sensitised	38.42	18.20	
Grass	LTP allergic	27.99	10	*p* = 0.006
	LTP sensitised	46.27	39.15	

**TABLE 2:** Sensitization to foods and aeroallergens.

Allergen	Total positive (% of those tested)		*p* value*
	LTP allergic	LTP sensitised	
Peach (SPT only)	78 (84%)	18 (31%)	<0.001
Almond	64 (71%)	50 (76%)	0.518
Hazelnut	59 (83%)	48 (80%)	0.648
Cor a 14	7 (11%)	19 (33%)	0.002
Cor a 9	22 (34%)	23 (43%)	0.318
Cor a 8	63 (82%)	23 (52%)	<0.001
Cor a 1	22 (54%)	23 (68%)	0.31
Macadamia nut	25 (54%)	18 (44%)	0.331
Peanut	64 (85%)	47 (81%)	0.508
Ara h 2	12 (10%)	37 (43%)	<0.001
Ara h 9	87 (95%)	26 (55%)	<0.001
Ara h 8	19 (41%)	23 (64%)	0.013
Pistachio	27 (39%)	35 (66%)	0.003
Cashew nut	15 (21%)	33 (52%)	<0.001
Ana o 3	5 (13%)	19 (44%)	0.001
Brazil nut	20 (29%)	20 (34%)	0.587
Ber e 1	3 (11%)	7 (17%)	0.461
Walnut	4 (40%)	40 (60%)	0.016
Jug r 1	61 (6%)	16 (40%)	<0.001
Jug r 3	3 (86%)	15 (79%)	0.454
Apple	44 (72%)	16 (73%)	0.967
Mal d 1	26 (53%)	9 (90%)	0.044
Mal d 3	10 (96%)	7 (88%)	0.331
Tomato	27 (81%)	11 (46%)	0.005
Birch	39 (55%)	65 (75%)	0.003
Grass	71 (69%)	101 (92%)	<0.001
Mixed tree	97 (65%)	30 (71%)	0.005
Mugwort	26 (54%)	19 (32%)	0.008
Plane tree	54 (68%)	25 (60%)	0.389

*Note*: *Pearson chi square.

**FIGURE 1:**
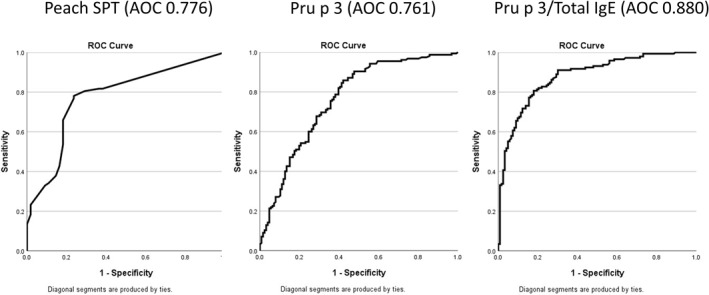
Receiver operating characteristics (ROC) curves to aid diagnosis. Comparison of ROC curves for Peach skin prick tests (SPT) extract, Pru p 3 and the Pru p 3/total IgE ratio.

Utilising the Pru p 3 and total IgE data, the ratio of Pru p 3 sIgE to total IgE was calculated to determine whether it was a more accurate diagnostic predictor than single tests alone. Using the mean values for Pru p 3:total IgE for each group and excluding patients with missing values for either total IgE or Pru p 3, the ratio for the LTP allergic group was 0.01065 (12.72:1193), compared to a ratio of 0.00171 (4.67:2275) for the LTP sensitised group (*p* < 0.00001). The ROC curve for Pru p 3:total IgE gave an Area Under the Curve (AUC) value significantly higher than all individual markers (AUC 0.880) (Figure 1). The point on the ROC curve corresponding to the best sensitivity and specificity was 0.007315, which had a sensitivity of 77% and a specificity of 84%; the positive and negative predictive values were 85.4% and 74.8% respectively.

To exclude an outlier effect, the calculations were repeated after removing individuals with total IgE levels greater than 10,000 KuA/L. Following this, the ratio for the LTP allergic patients was 0.01948 (12.72:616), compared to a ratio of 0.00217 (4.67: 1846) for LTP sensitised patients, with the difference remaining statistically significant (*p* < 0.00001). The ROC AUC remained superior to individual test results at 0.89. The point on the ROC curve corresponding to the optimal balance of sensitivity and specificity was 0.00733, with a sensitivity of 77.6% and a specificity of 84.3%, aligning with the AUC results that included the outliers.

### 3.3 Specific IgE and CRD results

**FIGURE 2:**
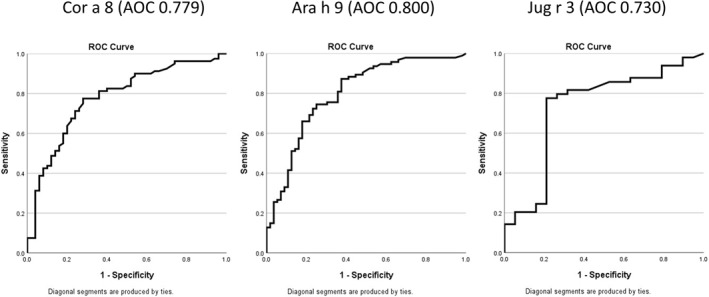
Receiver operating characteristics (ROC) curves for LTP from peanut, hazelnut, and walnut. Comparison of ROC curves for Cor a 8, Ara h 9 and Jug r 3.

In comparison with LTP sensitised patients, LTP allergic patients were significantly more likely to have a positive test for Cor a 8 (hazelnut LTP), Ara h 9 (peanut LTP), and specific IgE to walnut, tomato, and mugwort (Table 2). LTP sensitised individuals presented more frequently with sensitisation to Ara h 2, Cor a 14, pistachio nut, cashew nut, Ana o 3, Jug r 1, silver birch pollen and grass pollen (Table 2). There were also higher titres of IgE to Ara h 9 and Cor a 8 observed for those with LTP allergy (Table 1). To assess their diagnostic accuracy, ROC curves were performed for those diagnostic markers more frequently associated with a diagnosis of LTP allergy. The best results were for Ara h 9 (AUC 0.800), Cor a 8 (AUC 0.779), Jug r 3 (AUC 0.730), peach SPT (AUC 0.776), mugwort IgE (AUC 0.652) (Figures 1 and 2). Interestingly, the AUC for Pru p 3 was only a fair predictor of LTP allergy diagnosis (AUC 0.761) (Figure 1), and the level of total IgE showed an inverse association with the diagnosis. This might reflect the fact that those with a diagnosis of LTP‐allergic patients had a significantly lower level of total IgE than LTP sensitised patients.

### 3.4 Pru p 3 and correlation with other LTPs

A statistically significant linear correlation was observed between Pru p 3 and several LTP allergens, including Ara h 9 (*p* < 0.0001), Cor a 8 (*p* < 0.00001), Jug r 3 (*p* < 0.00001) Art v 3 (*p* < 0.00001) and mugwort IgE (*p* < 0.0001), but not for plane tree IgE or birch. For SPT, there was an extremely strong correlation between peach SPT and plane tree SPT (*p* = <0.00001), but no correlation between peach SPT and mugwort SPT (*p* = 0.239).

## 4 DISCUSSION

This is the first analysis of a large cohort of Pru p 3 sensitised patients in the United Kingdom to determine which tests best distinguish LTP allergy from sensitisation alone. In our cohort, LTP‐allergic patients had significantly higher levels of Pru p 3, and lower levels of total IgE, when compared to LTP sensitised patients. We also demonstrated that the ratio of Pru p 3:toal IgE may be a useful diagnostic marker in the UK population, and that testing with Ara h 9, Cor a 8, walnut‐specific IgE, tomato IgE and peach SPT could improve diagnostic accuracy.

There is already a good body of literature published regarding the best tests for the diagnosis of LTP allergy. Pastorello proposed that a cut off for Pru p 3 of 2.69 kUA/L could identify individuals with a higher risk of symptoms, although applying this to our data would mean that 49/157 (31%) patients with diagnosed LTP allergy had Pru p 3 levels below the cut off.^12^ Other studies have shown that there is an overlap between allergic and tolerant individuals, and Balsells‐Vives reported that 45% of their cohort with low Pru p 3 levels were considered to be allergic to LTP, suggesting that a Pru p 3 of 0.1 or lower could still be clinically relevant.^13^, ^18^ Some studies have already evaluated the specific food IgE:total IgE ratio as a tool in the diagnosis of food allergy.^19^, ^20^ It was demonstrated to have a significant ROC AUC of >0.5 in Pru p 3‐sensitized individuals from Antwerp, Belgium, but not in patients from Barcelona, Spain.^21^ However, in another large cohort of nearly 500 Spanish patients, Balsells‐Vives and colleagues also found that Pru p 3:total IgE ratios were higher in allergic versus tolerant individuals sensitised to Pru p 3.^13^ The ratio of Pru p 3 to total IgE was also demonstrated to be an important marker of severe symptoms including anaphylaxis in UK adults with LTP allergy.^7^

Our data showed that the AUC of the ROC curve for the Pru p 3:total IgE ratio was 0.886 and was the best discriminator between individuals only sensitised to Pru p 3 and those sensitised and allergic. More importantly for the clinicians' decision‐making, the PPV was 85%, meaning it could greatly facilitate diagnosis in 8/10 patients. Published studies have demonstrated that individuals sensitised to LTP could also be co‐sensitised to many other LTP,^11^ and also that sensitisation to a wider array of LTPs was more likely to be present in individuals who were symptomatic.^22^ A study on LTP allergy in the Mediterranean area reported that Jug r 3 was recognised by 82% of LTP patients, whereas Cor a 8 was only recognised by 56%, whereas in an Italian LTP allergic population, Ara h 9, Cor a 8 and Mal d 3 were best associated with clinical symptoms.^11^, ^15^, ^22^ Evidence from large cohorts suggests that those sensitised to five or more LTP were more likely to experience severe allergic reactions.^15^, ^23^

Our study identified that aside from the ratio of Pru p 3:total IgE, other useful diagnostic markers included Ara h 9, Cor a 8, specific IgE to walnut, tomato and peach SPT. Also, statistically significant correlations were observed between Pru p 3 and Ara h 9, Cor a 8 and Jug r 3, underlining their usefulness as additional markers in the diagnostic workup. Our data also demonstrated that specific IgE tests using whole protein extracts to peanut, hazelnut, almond, Macadamia nut, Brazil nut and apple were not effective in discriminating between those sensitised to Pru p 3 with LTP allergy and those who were sensitised but received an alternative diagnosis.

In addition to serum sampling, our data also reviewed the diagnostic efficacy of SPT testing with peach extract in individuals sensitised to Pru p 3. A review of the diagnosis and management of LTP allergy underlined the importance of ensuring that, where SPT extracts are used, they are known to contain Pru p 3.^9^ Our patients were all tested with the same extract (ALK Abelló, Horsholm, Denmark), which contains Pru p 3 and Pru p 7 (gibberellin‐regulated protein). The results showed that the SPT extract performed as well as Pru p 3 in the diagnosis of LTP allergy, and is a useful first line test, although it may be helpful to follow a positive SPT with testing to Pru p 3 to confirm reactions are due to LTP allergy rather than linked to gibberellin‐regulated proteins.^24^

Our data supports results from another UK study which suggests that peanuts and tree nuts were the most common food triggers reported by individuals with diagnosed LTP allergy^25^ Therefore, during the diagnostic work‐up for LTP allergy, a differential diagnosis of primary nut allergy needs to be excluded if nuts are reported to trigger reactions. A primary allergy to peanuts and some tree nuts characteristically starts in childhood and usually involves seed storage proteins,^26^ which are the best indicator of allergy to tree nuts in symptomatic patients.^27^ Thus, testing for the major seed storage protein in peanuts (Ara h 2), hazelnuts (Cor a 14), and walnuts (Jug r 1), can also be useful for symptoms of peanuts or tree nuts when LTP allergy needs to be ruled out. Some patients in our cohort had LTP allergy co‐existing with an often pre‐existing primary allergy to peanuts or tree nuts, and thus it is vital to understand which is predominant. For example, LTP‐allergic reactions may only occur in the presence of co‐factors or only in certain fruits or vegetables.

One confounding effect can be sensitisation to pollens, which can lead to erroneously positive tests to foods. PFS is a common food allergy affecting 2% of the population, and 4% in London.^1^ Thus, when reactions to fruits and tree nuts are reported, this must be considered to be a differential diagnosis; therefore, testing for pollen is important in order to help differentiate between PFS and LTP allergy in UK individuals sensitised to Pru p 3. Our results demonstrated that many of those diagnosed with LTP allergy were also sensitised to both birch and grass, suggesting that a diagnosis of LTP allergy cannot be ruled out in patients who are pollen sensitive In some populations, pollen proteins such as Art v 3, the mugwort LTP, are primary sensitising allergens,^28^, ^29^, ^30^ Previous UK data found a strong correlation between Pru p 3 and the LTPs in mugwort (Art v 3) and plane tree (Pla a 3).^4^ Our data did show a correlation between Art v 3 and Pru p 3, and that LTP‐allergic patients were more likely to be sensitised to mugwort.

The major limitation of this study is its retrospective nature. This meant that, apart from being tested to Pru p 3, there was variance in the other diagnostic tests performed, so the data available for every patient was not consistent. Also, diagnosis of LTP allergy was not confirmed by a FC. Another limitation is that only patients with a Pru p 3 greater than 0.35 were included in the study, whereas recent published studies suggest that patients may have clinically relevant LTP sensitisation if their levels are between 0.1 and 0.35. However, in mitigation, all patients included were not just sensitised to Pru p 3 but also had reported clinical symptoms to foods. We collected data on all patients tested to Pru p 3, and further studies could include an evaluation of those with levels above 0.1 kUA/L. The other limitation was the very high levels of total IgE, which may have skewed the ratio results; however, this has been addressed by evaluating the statistical significance by both excluding and including those with a total IgE greater than 10,000 IU. One reason might be that the group without LTP allergy had a higher level of reported atopic dermatitis than the group not diagnosed with LTP allergy (data not shown).

## 5 CONCLUSION

The diagnosis of LTP allergy in the UK is complex; sensitisation to Pru p 3 cannot be taken as a definitive marker of clinical LTP allergy in isolation unless the symptom history and foods involved are highly characteristic of LTP allergy. Analysis of our cohort yielded valuable information on diagnostic methods, including the use of the Pru p 3 sIgE:total IgE ratio as a diagnostic tool, which appears to be more accurate than peach SPT, Pru p 3 and other individual LTPs. Our data confirms the finding from Balsells‐Vives et al. that, in addition to a convincing history, the Pru p 3 sIgE:total IgE ratio supports a diagnosis of LTP allergy and may be especially helpful in cases of diagnostic doubt, or where an incidental finding of Pru p3/peach SPT positivity arises. In addition, testing for other LTPs, especially Ara h 9 abd Cor a 8, may provide further confirmation of a diagnosis of LTP allergy. On a practical level, our study results confirm that Pru p 3 alone is not diagnostic for LTP allergy and total IgE and results for other relevant LTP need to be taken into consideration. Future studies should aim to build on the available data to improve the accuracy of the diagnosis of LTP allergy. More studies are needed, especially from countries where LTP allergy is beginning to be recognised. Further characterisation of LTP allergy will enable better patient management, avoid unnecessary elimination diets and inappropriate prescription of adrenaline.

## AUTHOR CONTRIBUTIONS

**B. Olivieri**: Conceptualization; methodology; writing—original draft; investigation; formal analysis. **G. Scadding**: Writing—review and editing; project administration; supervision. **I. J. Skypala**: Conceptualization; writing—review and editing; methodology; formal analysis; project administration; supervision; resources; investigation.

## CONFLICT OF INTEREST STATEMENT

The authors declare no conflicts of interest.

## Data Availability

The data that support the findings of this study are openly available in Authorea at https://doi.org/10.22541/au.172979292.24357096/v1.
